# Rapid screening of ethylene glycol and diethylene glycol in raw materials and medicinal syrups using low-cost field deployable assays

**DOI:** 10.1038/s41598-025-26670-1

**Published:** 2025-12-03

**Authors:** Benediktus Yohan Arman, Isabelle Legge, John Walsby-Tickle, Tehmina Bharucha, Julia Gabel, Gesa Gnegel, Michael Deats, Sneha Banerjee, Robert Stokes, Hamid A. Merchant, Pavel Matousek, James McCullagh, Céline Caillet, Paul N. Newton, Nicole Zitzmann, Bevin Gangadharan

**Affiliations:** 1 Department of Biochemistry, University of Oxford, Oxford, OX1 3QU UK; 2 Kavli Institute for Nanoscience Discovery, University of Oxford, Oxford, OX1 3QU UK; 3 Department of Chemistry, University of Oxford, Oxford, OX1 3TA UK; 4 Medicine Quality Research Group, NDM Centre for Global Health Research, Nuffield Department of Medicine, University of Oxford, Oxford, OX3 7LG UK; 5 Mahidol-Oxford Tropical Medicine Research Unit, Faculty of Tropical Medicine, Mahidol University, Bangkok, 10400 Thailand; 6 Infectious Diseases Data Observatory, Centre of Tropical Medicine & Global Health, Nuffield Department of Medicine, University of Oxford, Oxford, OX3 7LG UK; 7 Pharmaceutical Institute, Eberhard Karls University Tübingen, Tübingen, Germany; 8 Central Laser Facility, Research Complex at Harwell, STFC Rutherford Appleton Laboratory, UKRI, Harwell Campus, Didcot, OX11 0QX UK; 9 Agilent Technologies LDA UK, Becquerel Avenue, Didcot, OX11 0RA UK; 10 Department of Bioscience, School of Health, Sport and Bioscience, University of East London, Water Lane, London, E15 4LZ UK; 11 Present Address: EXEINS Health Initiative, Jakarta, 12870 Indonesia

**Keywords:** Substandard, Medical products, Diethylene glycol, Ethylene glycol, Contamination, Rapid test, Falsified, Syrup, Renal failure, Drug regulation, Public health, Drug regulation, Drug safety, Pharmaceutics, Toxicology, Biochemical assays, Enzymes

## Abstract

There have been hundreds of child deaths due to contamination of medicinal syrups with diethylene glycol (DEG) and ethylene glycol (EG). Detection of DEG and EG is usually performed by gas chromatography, a method that is costly, laborious, time-consuming, and not readily available in many low- and middle-income countries (LMICs). Thin-layer chromatography is relatively lower cost and portable; however, as with gas chromatography, it requires time and trained personnel. Alternative rapid, low-cost and simple methods to determine DEG/EG contamination are needed. We tested the suitability of enzymatic, chemical and antibody-based assays to determine DEG/EG. Assays using alcohol dehydrogenase and aldehyde dehydrogenase alone as well as in combination with glycolate oxidase could determine EG in raw materials and at less than 0.1% m/m in some finished products. Saliva and breast milk alcohol test strips, containing alcohol oxidase and costing $1, could determine EG with a detection limit of 0.5 to 2% m/m in under 2 minutes. Disposable breathalysers, costing only $1, could determine both DEG and EG from other alcohols in only 10 seconds. The methods described provide simple, rapid and low-cost alternatives to help determine DEG and EG contamination in pharmaceutical supply chains to help prevent deaths where equipment and trained human resources are limited.

**Supplementary Information:**

The online version contains supplementary material available at 10.1038/s41598-025-26670-1.

## Introduction

Substandard and falsified (SF) medical products are a global phenomenon but are neglected public health threats. Although they are more prevalent in low- and middle-income countries (LMICs), they are often difficult to detect and control due to under-resourcing of some national medicine regulatory authorities (NMRAs)^1–3^. The substandard medical products do not meet their quality standards and/or quality specifications as a result of poor manufacturing practices or are degraded due to inappropriate storage on supply chains^1,4^. In contrast, falsified medical products ‘deliberately and fraudulently misrepresent their identity, composition or source’^1,4^ and pose a serious risk to public health.

**Fig. 1:**
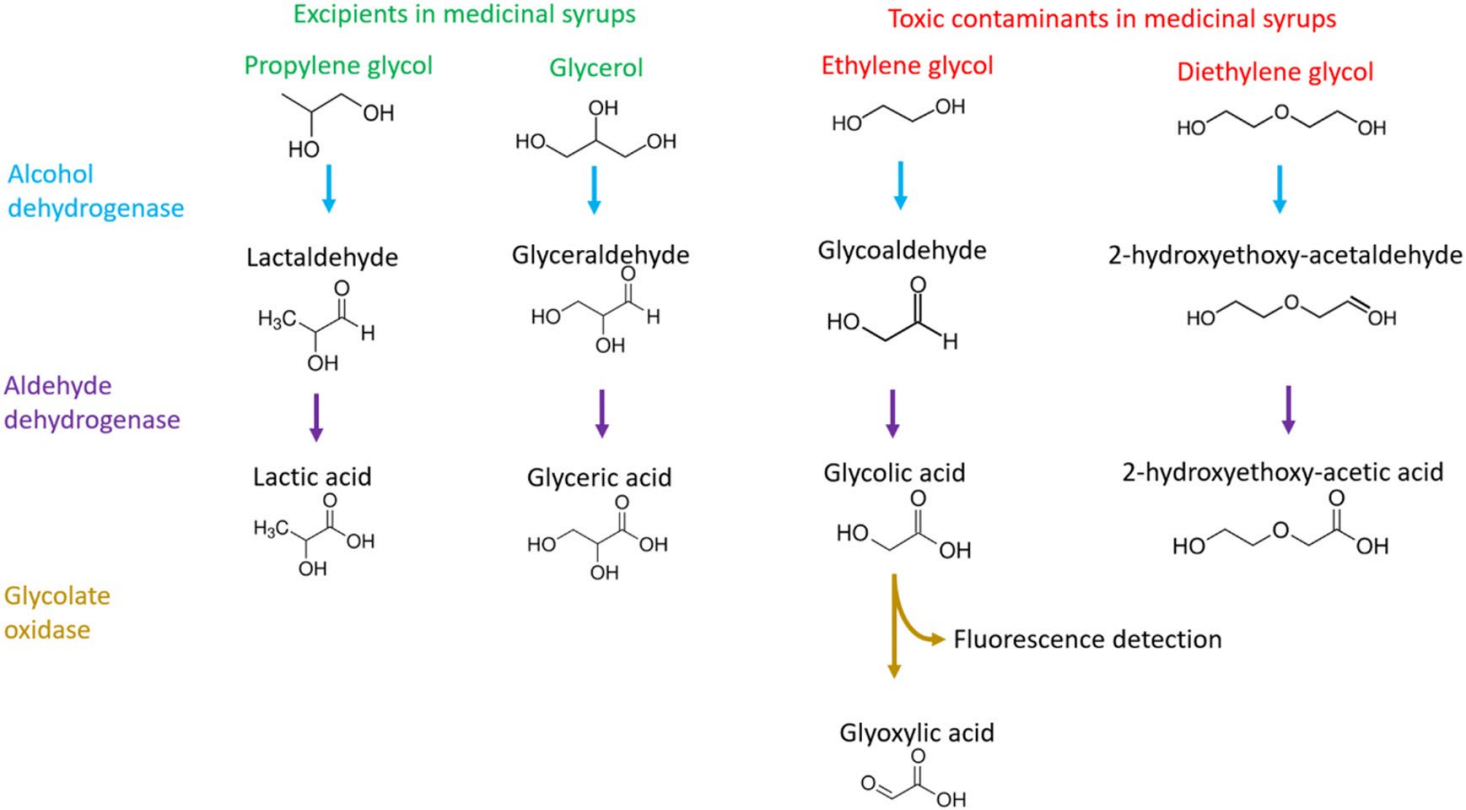
The metabolic pathways of PG and glycerol used as excipients in syrups and DEG/EG, including the use of glycolate oxidase for the detection of glycolic acid. To simplify this pathway, steps with NAD^+^ and NADH have been omitted.

Medicinal syrups often contain excipients such as propylene glycol (PG) and glycerol as a base to formulate the liquid dosage forms. These must be of pharmaceutical grade so that they meet stringent purity standards approved for human consumption. It is vital to limit the presence of toxic impurities, such as diethylene glycol (DEG) and ethylene glycol (EG), to negligible safe levels. DEG and EG are industrial solvents often used in antifreeze and vehicle brake fluid and should never be used as excipients in medicinal syrups due to their known toxicity. Upon ingestion they are metabolised to the toxic acids 2-hydroxyethoxyacetic acid (HEAA), glycolic acid and calcium oxalate which can lead to neurological damage, renal failure, metabolic acidosis, hyperosmolality and death^5^. These toxic metabolites originate from the enzymatic reactions involving alcohol dehydrogenase and aldehyde dehydrogenase^5,6^ (Fig. 1).

DEG and EG contamination in medical products may result from inadvertent or deliberate mislabelling of barrels since both industrial solvents are much less expensive than that of pharmaceutical-grade PG and glycerol. Moreover, industrial-grade PG and glycerol are also widely available and are less expensive than their pharmaceutical grade counterparts. These should not be used in formulating medicines, as impurities such as DEG and EG are present at much higher levels in industrial grade PG and glycerol than their pharmaceutical counterparts^7^. The ingestion of DEG can lead to serious, potentially lethal, health effects due to metabolic acidosis caused by the metabolite HEAA^7^. The metabolites from EG, glycolic acid and oxalic acid, are toxic and contribute to significant metabolic acidosis^8–11^. The first reported case of contaminated syrup intoxication occurred in 1937 in the United States of America with the antibiotic product Elixir Sulfanilamide^12^. Multiple outbreaks have since occurred in different parts of the world leading to several hundred deaths due to DEG/EG mediated toxicity^5^. From October 2022 to October 2025, over a dozen medical product alerts were issued by the WHO and medicine regulators for more than 40 over-the-counter (OTC) liquid medicines contaminated with DEG and/or EG in The Gambia^13^, Indonesia^14^, Uzbekistan and Cambodia^15^, the Federated States of Marshall Islands and Micronesia^16^, Cameroon^17^, the Republic of Iraq^18^, the Maldives and Pakistan^19,20^, Thailand^21^, India^22,23^, Kenya, Nigeria, Rwanda, South Africa, Tanzania, and Zimbabwe^24^. It was estimated there were at least 300 fatalities in children worldwide reported in 2022 associated with DEG/EG toxicity liquid medicines^25^. In The Gambia, the adulteration of antihistamine, cough and cold syrups led to the deaths of 70 children, while in Indonesia, the Indonesian Ministry of Health reported more than 324 cases of acute kidney injury leading to 199 child deaths due to the unsolicited use of EG/DEG in liquid medicines^26,27^. More recently, more than 24 children died in India after consuming syrup contaminated with DEG^28^.

In 2022, the Indonesian Food and Drug Authority (Badan Pengawas Obat dan Makanan Republik Indonesia/BPOM RI) identified barrels labelled as PG but were contaminated with 4.69 to 99.09% EG^29^. In another report, BPOM RI found cases of PG contaminated with 33.46% EG and 5.94% DEG. In addition, 1.28 to 443.66 mg/mL (equivalent to 0.13 to 44.37% m/m) of EG and DEG contamination was detected in finished syrup products^30^. The minimum lethal dose of DEG/EG intoxication in humans can vary in individuals due to inter-subject and population variability in metabolic enzyme expression and metabolic capacity which makes it complicated to ascertain a simplified safety limit^31^. Medicine regulators require all syrup manufacturers to test all barrels of PG and glycerol to assure the absence of DEG/EG impurities or contamination at less than 0.1% m/m DEG and EG, the maximum permissable limits set for pharmaceutical grade raw ingredients^32^. However, there are reports of failure to adhere to manufacturing quality control standards^33^.

The compendial recommendation for the detection and quantitation of DEG/EG in syrups is by gas chromatography (GC), which is costly, laborious, time-consuming and often not available in many LMICs^34–37^. The use of thin-layer chromatography (TLC) provides a lower-cost and portable method to screen DEG/EG in medical products^38^. However, TLC has failed to detect contaminants in cough syrup^39^ and, although is less expensive and easier to perform than GC, still requires personnel with significant laboratory skills^37,40^. The WHO has drafted a chapter for The International Pharmacopoeia which includes protocols for both TLC and GC that involves over a dozen steps by its virtue for each method including preparing both syrups and reference solutions in alcohols with volumetric flasks^40^. Although TLC is a lower-cost approach compared to GC, the method requires several consumables including silica gel TLC plates, a chromatographic tank, a hairdryer, solvents (which are toxic, flammable, corrosive and hazardous), a visualisation solution containing potassium permanganate (which is very toxic, corrosive and harmful) and a laboratory fume hood. Alternative rapid, low-cost, portable and simpler methods to determine DEG/EG contamination are therefore desirable, which do not require harmful chemicals/solvents or as many consumables as TLC and GC, so that they could be easily performed by inspectors even outside of a scientific laboratory at various points in a supply chain. To our knowledge, the only low-cost and portable test which can successfully differentiate glycerol from DEG is by using an mbira and a mobile phone^41^. However, the mbira has not been tested for PG and EG and therefore it is unknown if this could have helped to identify the barrels in Indonesia and Pakistan which were recently labelled as PG but had 96–100% EG^19,42^. Furthermore, the limit of detecting DEG in glycerol using an mbira has not been investigated and it is unlikely that it would be able to detect low levels of DEG or EG near the toxic threshold levels.

As a low-cost approach, we initially replicated how EG is metabolised in the body by converting it using alcohol dehydrogenase and aldehyde dehydrogenase to form glycolic acid which could then be detected using glycolate oxidase and a substrate (Fig. 1). By analysing enzyme kinetics prior to the addition of glycolate oxidase, we observed that the conversion of EG was far higher than the other alcohols. We hypothesised that EG may also convert quickly with alcohol oxidase and therefore could be used in a low-cost colorimetric rapid test. This led us to test a simple, rapid and low-cost assay to determine EG presence by repurposing rapid diagnostic tests, commonly used for detecting alcohol in human saliva and breast milk, which use alcohol oxidase and a colorimetric substrate. For DEG, we investigated if a polyethylene glycol (PEG) antibody, which recognises the polyether chain of PEG, could preferentially recognise the ether in DEG. Disposable breathalysers contain an oxidiser that changes colour with alcohols and by exploring whether the alcohols oxidise at different rates, we could successfully use them to differentiate both DEG and EG from the other alcohols rapidly and at very low-cost. We have also written a step-by-step protocol for both the alcohol strips and breathalysers which can be used by inspectors to determine EG and DEG in both raw materials and finished products.

Unlike GC and TLC, these novel methods do not require any harmful chemicals or solvents and can be used as rapid screening tests in field settings with minimal training. The tests could also provide useful preliminary indicators of DEG and EG prior to using more selective and sensitive reference testing.

## Results

### Determining EG in raw materials using enzymatic assays

**Fig. 2:**
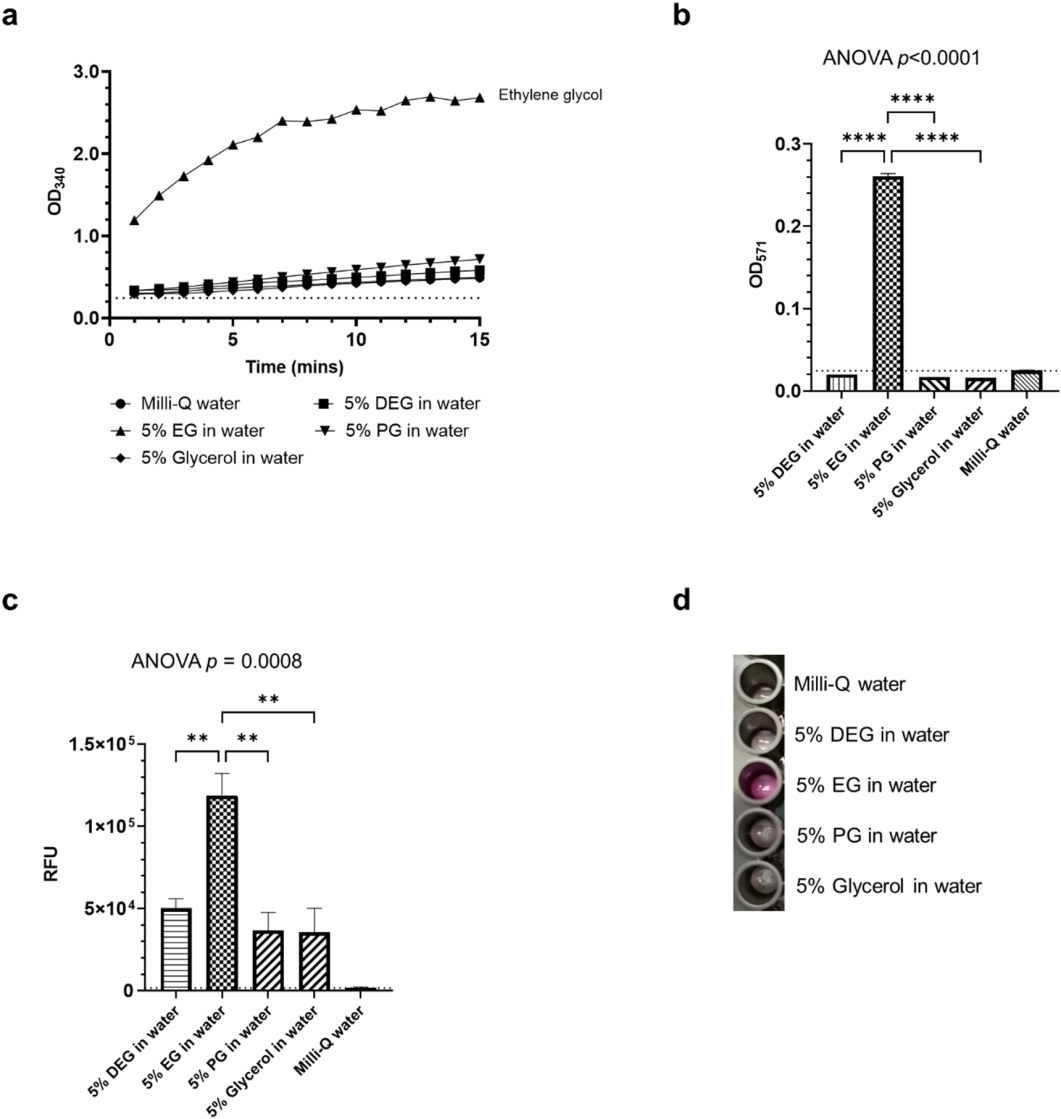
Determining EG using enzymatic assays. (**a**) Using alcohol dehydrogenase and aldehyde dehydrogenase, EG was found to convert at a far higher rate than the other alcohols. NADH was measured at 340 nm over 15 min. The dotted line shows the blank absorbance limit. (**b**) By additionally using glycolate oxidase to detect glycolic acid, EG could be determined more specifically. End-point absorbances were measured at 571 nm. (**c**) The detection of glycolic acid in alcohol samples, measured as the relative fluorescence unit (RFU). (**d**) Successful identification of EG simply by visual observation of the oxidised fluorogenic substrate as pink colour without the need for a plate reader. No pink colour was observed for the other alcohols. Error bars show the standard deviations of two replicates. Ordinary one-way ANOVA with Dunnett’s multiple comparison. **p < 0.01; ****p < 0.0001.

**Fig. 3:**
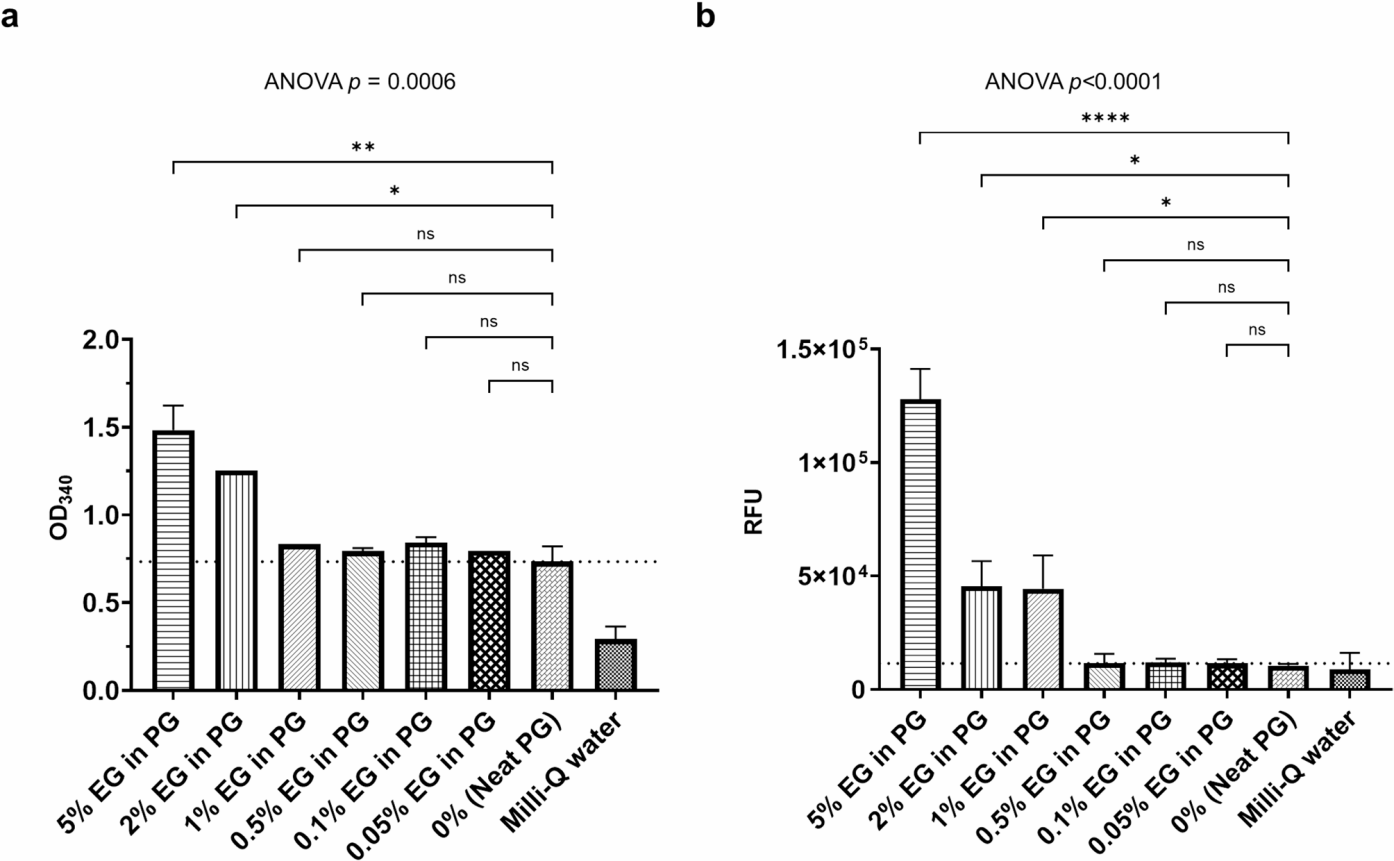
Determining EG in PG using enzymatic assays. PG was spiked with different percentages of EG. (**a**) End-point 340 nm absorbance readings of NADH after using alcohol dehydrogenase and aldehyde dehydrogenase. (**b**) Relative fluorescence unit (RFU) readings after additionally using glycolate oxidase in the glycolic acid assay. Milli-Q water was used as a no-matrix control. The dotted line shows the limit of neat PG. Error bars show the standard deviations of two replicates. Ordinary one-way ANOVA with Dunnett’s multiple comparison. ns, not significant; *p < 0.05; **p < 0.01; ****p < 0.0001.

Neat EG was successfully differentiated from the other neat alcohols (glycerol, PG and DEG) by diluting each separately to 5% in water followed by using alcohol dehydrogenase, aldehyde dehydrogenase and a plate reader to record the formation of NADH at 340 nm (Fig. 2a). By additionally using glycolate oxidase, a fluorogenic substrate, and recording the fluorescence with a plate reader, it was possible to more specifically determine EG presence and differentiate it from other alcohols by analysing end-point absorbance (Fig. 2b) and relative fluorescence units (Fig. 2c). More importantly, it was possible to successfully identify EG simply by visual observation of the oxidised fluorogenic substrate as pink colour (Fig. 2d) without the need for a plate reader. Different concentrations of EG spiked into PG were also tested and EG could be confidently determined down to 2% m/m using alcohol dehydrogenase and aldehyde dehydrogenase (Fig. 3a, Supplementary Fig. 1a); and 1% m/m when additionally tested using the glycolic acid assay (Fig. 3b, Supplementary Fig. 1b).

### Determining EG in medicinal syrups using enzymatic assays

**Fig. 4:**
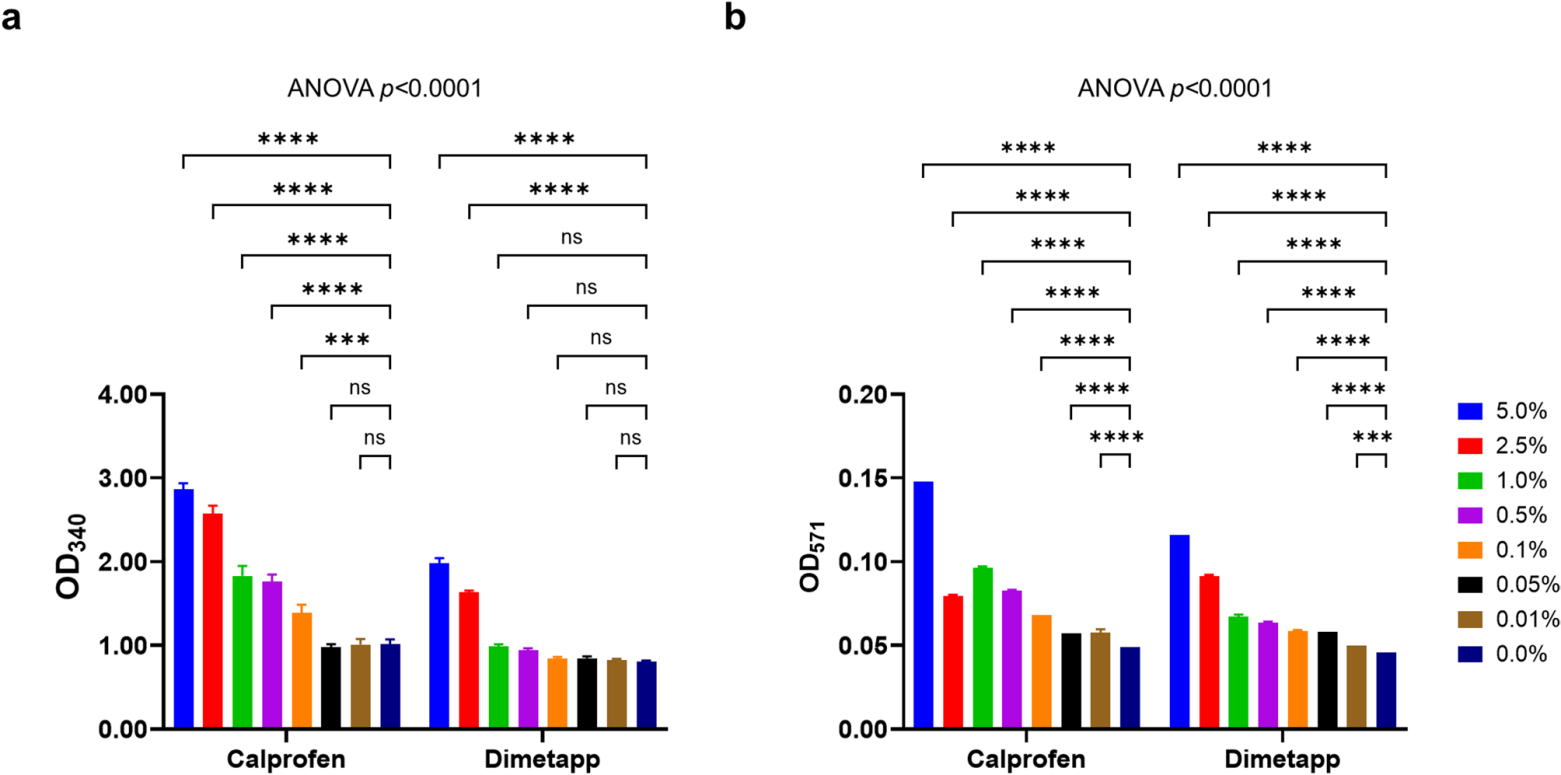
Enzymatic assay results for two paediatric syrups (Calprofen and Dimetapp) spiked with different percentages of EG. (**a**) End-point 340 nm absorbance readings of NADH after using alcohol dehydrogenase and aldehyde dehydrogenase. (**b**) End-point 571 nm absorbance readings after additionally using glycolate oxidase in the glycolic acid assay. Error bars show the standard deviations of two replicates. Two-way ANOVA with Dunnett’s multiple comparison. ns, not significant; ***p < 0.005; ****p < 0.0001.

EG was determined down to 0.1% and 0.5% m/m in EG-spiked paediatric Calprofen and Dimetapp syrups, respectively, using alcohol dehydrogenase and aldehyde dehydrogenase (Fig. 4a, Supplementary Fig. 2a). By additionally using the glycolic acid assay, significant differences in fluorescence were detected down to 0.01% m/m for both paediatric syrups (Fig. 4b), Supplementary Fig. 2b), although only small absorbance differences were observed in the syrups with EG spiked below 0.1% m/m. The assays did not perform well to determine EG presence in some ethanol-containing syrups suitable for teenagers and adults (Beechams and Benylin Chesty), but they did work successfully for ethanol-containing Covonia down to below 0.1% m/m (Supplementary Fig. 3).

### Alcohol test strips to determine EG in raw materials

**Fig. 5:**
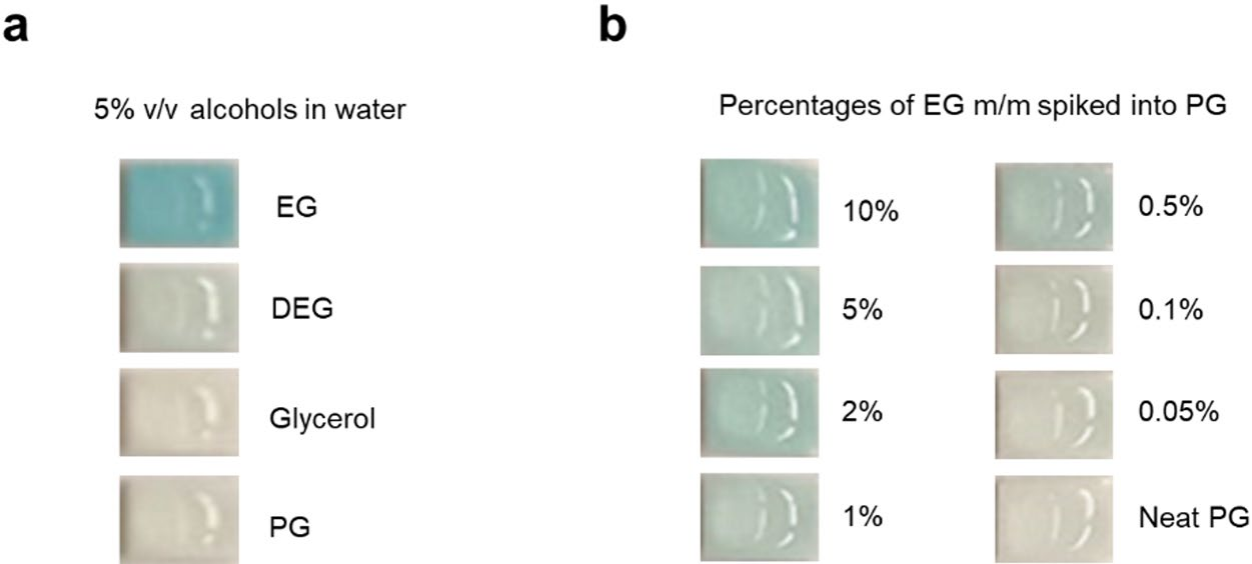
Determining EG using Surescreen alcohol saliva strips. Photos show zoomed-in images of the pad on the strips. (**a**) Successful conversion of EG with the alcohol oxidase in the pad of the strip (blue colour) and differentiation from glycerol, PG and DEG using 5% v/v of the alcohols in water. (**b**) Different percentages of EG spiked into neat PG. Limit of detection ~ 0.5% EG m/m.

Five different brands of alcohol test strips designed to detect alcohol in saliva and breast milk were tested and all could successfully and rapidly (within 2 min) differentiate EG from glycerol and PG (Supplementary Fig. 4, Fig. 5a). These strips did not show any colour change with undiluted EG and only worked with EG diluted in water with the optimal dilution being 5% v/v EG in water (data not shown). A sixth brand (Wondfo One Step Alcohol Saliva Test, Guangzhou, China) showed similar results (data not shown). The Surescreen alcohol saliva strip could also determine EG presence down to 0.5% m/m when spiked into PG (Fig. 5b). A slight, but hard to observe, colour change was observed for DEG (Fig. 5a).

### Alcohol strip tests to determine EG in medicinal syrups

**Fig. 6:**
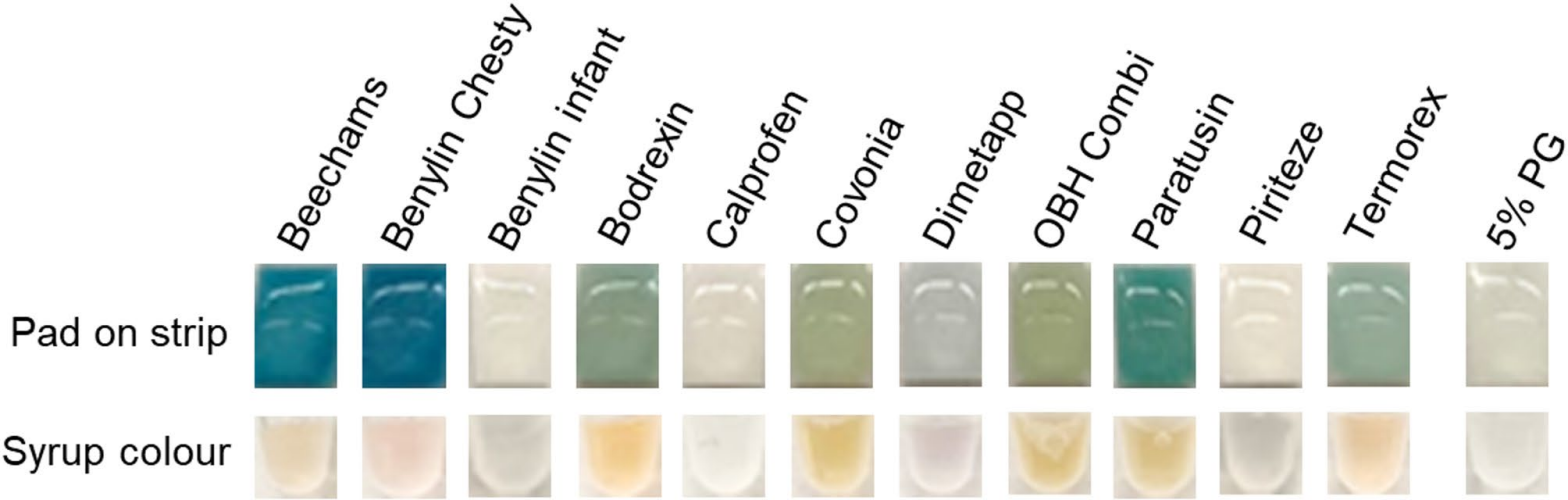
Investigating if ethanol and the colour of syrups interfere with the alcohol saliva strip test results. *Upper* row: Eleven neat medicinal syrups were applied to the pad of the strips and compared to a 5% v/v PG solution in water. Photos show the zoomed images of the pads on the strips. A blue colour on the pad was observed for Beechams, Benylin Chesty, Paratusin (all ethanol-containing) whereas alcohol-free paediatric syrups (Benylin Infant and Piriteze) and alcohol-containing paediatric syrups (Calprofen) did not show any colour change. There was no blue development colour on the pads for some syrups although they were coloured due to the colour of the syrups (Covonia, OBH Combi and Dimetapp). *Lower* row: The syrups were pipetted into transparent plastic tubes and the colours were noted. The photos show zoomed-in images of the bottom of the tubes. Coloured syrups, such as Covonia and Dimetapp, contribute to the colour observed on the pads of the strips and therefore could interfere with the visualisation of any blue colour if EG were to be present in similarly coloured syrups.

Similarly to raw materials, EG presence was detected in medicinal syrups best when diluted 5× in water before being applied to the pad (dilution optimisation data not shown). No change in the colour of the saliva strips was observed for Benylin Infant and Piriteze syrups, both of which are alcohol-free paediatric syrups (Fig. 6; Table 1). Dimetapp, also known not to be formulated with ethanol, did not show a blue colour change, but the pad was slightly coloured due to the syrup colour. Calprofen and Covonia, both stated to contain traces of ethanol, showed no blue colour on the pad of the strips, although for Covonia, the pad was coloured due to the syrup colour. All other syrups stated to contain ethanol (Beechams, Benylin Chesty and Paratussin) generated a blue colour on the pad of the strips. Visualisation of the blue colour change was more noticeable in colourless syrup samples (Fig. 6).

**Table 1:** Medicinal syrups bought from registered pharmacies in the UK, USA, and Indonesia.

Brand	Manufacturer	Intended use	Active Pharmaceutical Ingredients	Excipients
Beechams all in one	GlaxoSmithKline, Brentford, UK	Adults and children aged 16 years and over	Paracetamol, guaifenesin, phenylephrine hydrochloride	**Ethanol (19% v/v)**, sodium propylene glycol, sorbitol, glycerol
Benylin chesty coughs non-drowsy	McNeil, High Wycombe, UK	Adults and children aged 12 years and over	Guaifenesin, levomenthol	Sucrose, liquid glucose, **ethanol**, glycerol, sodium citrate, saccharin sodium, citric acid monohydrate, sodium benzoate
Benylin infant’s cough syrup	McNeil, High Wycombe, UK	Children aged 3 months to 5 years	Glycerol	Maltitol liquid, Sodium, Sodium benzoate, Propylene glycol
Bodrexin flu & batuk PE	PT. Tempo Scan Pacific, Tbk., Bekasi, Indonesia	Children below 12 years	Paracetamol, phenylephrine hydrochloride, guaifenesin, bromhexine hydrochloride, chlorphenamine maleate	Information not available in public domain
Calprofen	McNeil, UK	Babies and children aged 3 months to 12 years	Ibuprofen	Glycerol, xanthan gum, polysorbate 80, flavouring agent (contains propylene glycol and **ethanol**), maltitol, saccharin sodium, citric acid monohydrate, sodium methyl hydroxybenzoate, sodium propylhydroxybenzoate
Covonia dry and tickly cough linctus	Thornton and Ross, Huddersfield, UK	Adults and children aged over 1 year	Honey, capsicum tincture, menthol, peppermint oil, anise oil, liquorice extract	Glycerol, **ethanol** (in capsicum tincture), citric acid, glucose, flavouring agent (contains ethanol), propylene glycol
Dimetapp cold and cough	Foundation Consumer Brands, LLC., USA	Children 6 to under 12 years, children 12 years and older and adults	Brompheniramine maleate, dextromethorphan HBr, Phenylephrine HCl.	Anhydrous citric acid, glycerin, propylene glycol, sodium benzoate, sodium citrate, sorbitol solution, sucralose
OBH Combi Anak Batuk plus flu	Combiphar, Bandung, Indonesia	Children aged 2 to 12	Paracetamol, succus liquiritiae, ammonium chloride, pseudoephedrine hydrochloride, chlorphenamine maleate	Information not available in public domain
Paratusin	PT. Darya Varia Laboratories, Tbk., Bogor, Indonesia	Adults and children aged 2 to 12	Paracetamol, pseudoephedrine hydrochloride, noscapine, chlorphenamine maleate, guaifenesin, succus liquiritiae	Contains **ethanol (10% v/v)**, other excipients are not stated in the packaging or product insert.
Piriteze children’s hayfever & allergy syrup (GSL)	Haleon, Weybridge, Surrey, UK	Children 2 years and above	Cetirizine hydrochloride	Glycerol, propylene glycol, Liquid sorbitol (non crystallising) (E420), methyl parahydroxybenzoate (E218), propyl Parahydroxybenzoate (E216), sodium acetate, acetic acid, saccharin sodium, banana flavour, purified water
Termorex plus flu dan batuk	PT. Konimex, Sukoharjo, Indonesia	Children aged 2 to 12	Paracetamol, pseudoephedrine hydrochloride, guaifenesin, chlorphenamine maleate	Information not available in public domain.

Ethanol is shown in bold to highlight the syrups containing this excipient.

### Alcohol strip tests to determine EG spiked into infant medicinal syrups

**Fig. 7:**
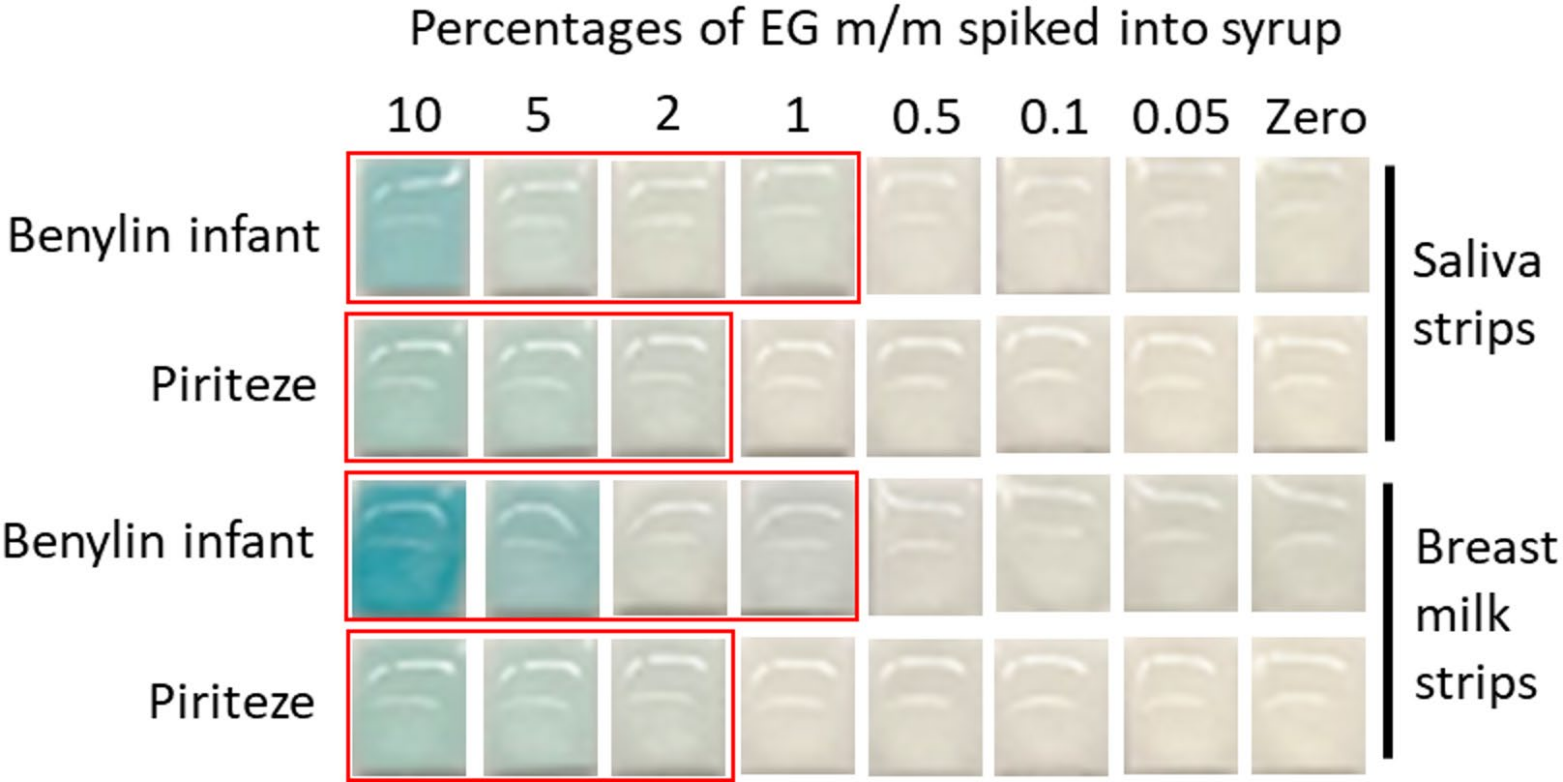
Determining EG spiked into medicinal syrups. Zoomed-in images of the pads on the strips are shown. The Surescreen alcohol saliva and Frida mom breast milk strips could determine the presence of EG down to 1% and 2% m/m for Benylin infant and Piriteze, respectively. The red boxes show where the blue colour on the pad could be seen when visually observed.

Benylin infant and Piriteze syrups were spiked with various concentrations of EG. Alcohol test strips for both saliva and breast milk were successfully used to determine EG presence (Fig. 7). The lowest detectable levels of EG in the spiked syrups, for both alcohol strips were 1% and 2% m/m EG in Benylin infant and Piriteze syrups, respectively.

### Breathalysers to help determine DEG and EG

**Fig. 8:**
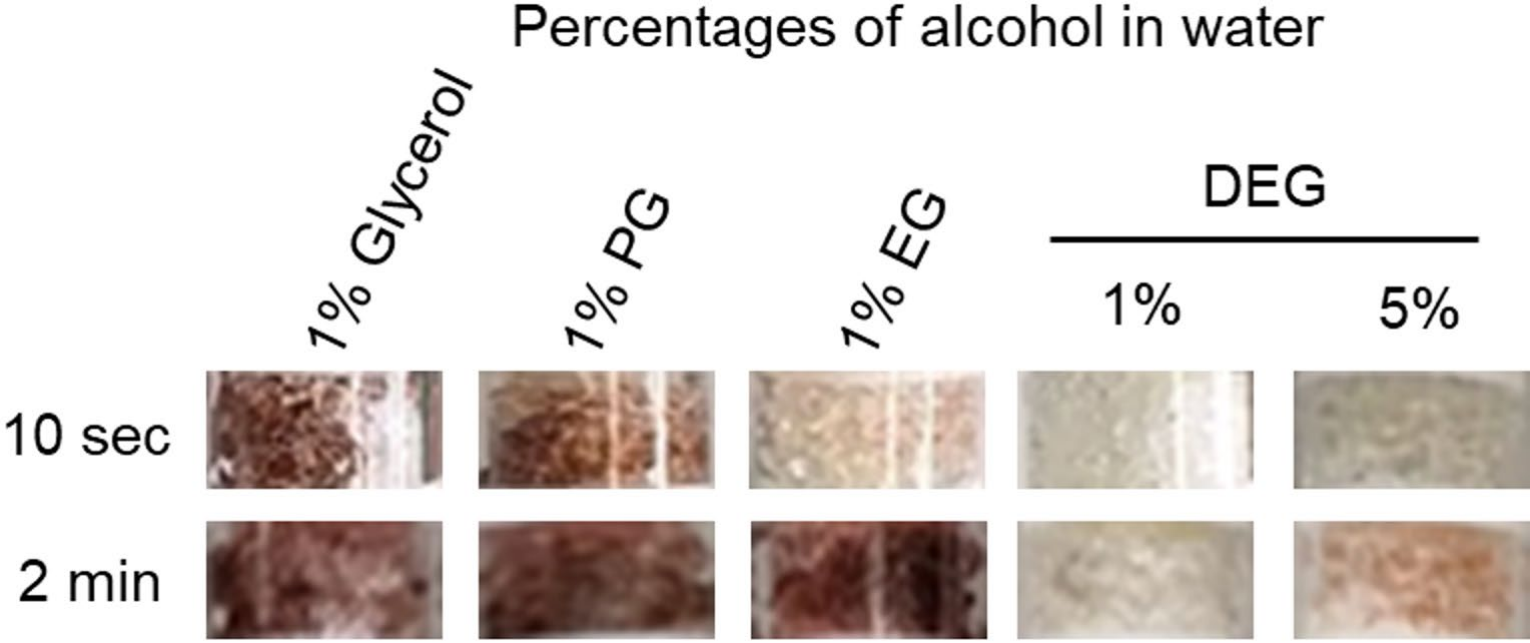
Disposable breathalysers can successfully indicate if glycerol or PG raw materials have been substituted with DEG or EG. Zoomed-in images of the crystals seen through the viewing window of the breathalyser are shown. All alcohols were diluted to 1% v/v except for DEG which was additionally tested at 5% v/v. In only 10 s, glycerol and PG used in raw materials changed the crystals in the breathalyser from white to dark brown whereas DEG showed no change in colour and EG showed a very faint pink colour. There was also no change in the colour of the crystals after 10 s when DEG was tested at the 5× higher concentration. At 2 min, there was still no colour change for DEG. The crystals changed to dark brown for EG suggesting that the white crystals do oxidise EG but at a slower rate compared to glycerol and PG, both of which remained dark brown at 2 min. For DEG, there was a slight change in the colour of the crystals to pink after 2 min when tested at the 5× higher concentration although was still a lot less intense than glycerol and PG when tested after 10 s.

Disposable breathalysers could help to determine both DEG and EG by observing the rate of colour change of the crystals after the addition of the samples. Using a 1% v/v dilution in water, both glycerol and PG samples rapidly changed the colour of the crystal in test tubes from white to dark brown from as early as 10 s whereas DEG showed no colour change at all and EG showed a very pale pink colour (Fig. 8). The diluted EG sample turned dark brown after two minutes whereas the DEG sample did not change the crystal colour even after two minutes (Fig. 8). The breathalysers worked best with the alcohols diluted to 1% v/v in water. When using a higher 5% v/v DEG in water, a faint pink colour change was observed after two minutes (Fig. 8) and the pink colour intensity was considerably weaker than the dark brown observed for the glycerol and PG samples even when used at only 1% v/v. The breathalysers were tested using 0.1% v/v alcohols in water and they were also able to successfully differentiate DEG from the other alcohols (Supplementary Fig. 5).

### PEG ELISA for DEG determination

The competitive PEG ELISA showed very weak preferential binding of DEG due to the lowest absorbance compared to the other three alcohols, although the difference was very minor and not reliable enough to confidently differentiate DEG from the other alcohols (Supplementary Fig. 6).

## Discussion

GC and TLC are the two recommended tests for the detection of DEG and EG in raw materials and formulated medicinal syrups^40^. In this study, we have explored alternative rapid, low-cost and portable methods to determine DEG and EG presence in both raw materials and finished products. These tests use enzymatic, chemical and antibody-based assays which, unlike GC and TLC, do not require any sophisticated laboratory facilities, expensive reagents or harmful chemicals.

The alcohols, glycerol and PG, used in syrups are converted by alcohol dehydrogenase and aldehyde dehydrogenase to acids which are safely cleared by the body. While DEG and EG themselves are not toxic, they are converted by the same two enzymes into very toxic metabolites (Fig. 1). We used alcohol dehydrogenase and aldehyde dehydrogenase enzymes in vitro to replicate the metabolism which occurs in vivo for individuals who have inadvertently consumed these alcohols. In the case of EG, it can be metabolised to glycolic acid for which an oxidase, glycolate oxidase, exists allowing it to be detected simply with a chromogenic or fluorogenic substrate (Fig. 2b–d). We are unaware of an oxidase for HEAA or any other metabolites for DEG. Also, an oxidase for HEAA may not help since we observed little conversion of DEG with alcohol dehydrogenase and aldehyde dehydrogenase (Fig. 2a). Therefore, we did not explore enzymatic assays for DEG. We successfully demonstrated that alcohol dehydrogenase and aldehyde dehydrogenase could be used to determine EG presence and differentiate it from other glycols (Table 2) and, when used in combination with a glycolic acid assay, could determine EG presence as low as 1% m/m in PG (Fig. 3b) and down to < 0.1% m/m in some formulated medicinal syrups (Fig. 4b). The glycolate oxidase used in this study was expected to be specific for glycolic acid, an alpha hydroxy acid. Although, the additional use of this enzyme did not work well with teenage/adult syrups containing ethanol, they did work successfully for ethanol-containing Covonia down to below 0.1% m/m (Supplementary Fig. 3). This is most likely due to Covonia having low levels of ethanol (Fig. 6) since it is suitable for children over 1 year old in addition to teenagers and adults. Ethanol was not expected to interfere in the assay since it would have been converted to acetaldehyde with alcohol dehydrogenase and then oxidised by aldehyde dehydrogenase to acetic acid which is not an alpha hydroxy acid and not an expected substrate for glycolate oxidase.

**Table 2:** Summary of the assays repurposed to help determine ethylene glycol (EG)/diethylene glycol (DEG).

Assay	Intended use	Repurposed for DEG and/or EG	LoD (% m/m in matrix)	Proposed use	Advantages	Limitations
Ethanol assay (absorbance)	Ethanol	EG	2.0% (in PG); 0.1–0.5% (in syrup)	Raw materials and ethanol-free paediatric syrups	Commercially available kit. Good sensitivity.	Requires a spectrophotometer. Cannot be used with syrups containing ethanol.
Glycolic acid assay (fluorescence)	Glycolic acid	Glycolic acid from EG	1.0% (in PG); 0.1% (in syrup)		Commercially available kit. Very sensitive detection using fluorometry.	Requires instrument with fluorescence measurement capability. Cannot be used with syrups containing ethanol.
Alcohol strip tests (saliva or breast milk)	Ethanol	EG	0.5% (in PG); 1.0–2.0% (in syrup)		Rapid, simple, very low-cost ($1), device-free. Reasonably good sensitivity. Commercially available.	Cannot be used with syrups containing ethanol.
Alcohol breath test	Ethanol	DEG and EG	~ 0.1 and 1.0%, respectively (in water)	Raw materials only	Rapid, simple, very low-cost ($1), device-free. Commercially available.	Relies on a negative result for DEG and slower reaction for EG and therefore can only be used for raw materials.
PEG ELISA	PEG	DEG	Only 5.0% tested (in water)	Note: Custom antibody or aptamer required for further development	Commercially available kit.	Designed to detect PEG. Poor results for DEG.

DEG, diethylene glycol; EG, ethylene glycol; ELISA, enzyme-linked immunosorbent assay; LoD, limit of detection; PG, propylene glycol; PEG, polyethylene glycol.

These assays could potentially be used in NMRA labs, hospitals, or medicine testing laboratories. Although a plate reader is required to measure the absorbance of NADH at 340 nm, we have demonstrated that EG could also be determined without the need for a plate reader since the reduced fluorescent substrate is visible as pink by the naked eye (Fig. 2d). It may be possible for this enzymatic test to be developed into a low-cost and rapid pad-based strip test using a cocktail of four enzymes (alcohol dehydrogenase, aldehyde dehydrogenase, glycolate oxidase and a peroxidase) and a chromogenic substrate such as tetramethylbenzidine to produce a colour change visible to the naked eye. While this should work for raw materials, a potential problem is the testing of finished products since some syrups are already coloured (Fig. 6) which may limit the standardised colour change. To overcome this problem, a fluorogenic substrate could be used instead since syrups are free from fluorescent excipients and this approach potentially has the advantage of greater sensitivity. Rapid tests using florescence have successfully been visualised simply using a low-cost UV torch (e.g. Hough COVID-19 Home test, Burleigh West, Australia). Due to the resource constraints, we were unable to explore the possibility of developing such a rapid test, however, this is a potential next step.

Conversion of EG was far higher than the other alcohols when using only alcohol dehydrogenase and aldehyde dehydrogenase (Fig. 2a). Since EG conversion using these enzymes was more like ethanol than the other alcohols, we hypothesised that EG may also convert faster than the other alcohols with alcohol oxidase. This led us to evaluate alcohol rapid test strips which use alcohol oxidase, a peroxidase and a chromogenic substrate which turns blue in the presence of alcohol. By repurposing these alcohol test strips, we were able to demonstrate that EG can not only be rapidly determined but can also be easily differentiated from glycerol and PG, both of which yielded negative results (Fig. 5a). This successfully worked with three different brands of saliva strips and three brands of breast milk strips (five shown in Supplementary Fig. 4) suggesting that this approach may work with multiple brands of alcohol rapid test strips for testing raw materials. These rapid (2 min) and inexpensive (less than $1) test strips do not require any scientific instrumentation and are easy to use with minimal training since they are designed for public use. We include a step-by-step protocol sheet on how an inspector could use these test strips to determine EG presence in both raw ingredients and finished products (Supplementary Protocol). Currently, interpretation is performed by visual inspection of the pad where any colour change on the pad to blue would indicate EG contamination. The test could be improved using a mobile phone app to take photographs of the breathalysers/strips and determining the possible DEG/EG contamination via an integrated algorithm without the need for visual inspection.

The strips have the enzymes alcohol oxidase and peroxidase in the pad and are designed for testing the presence of alcohol in saliva and breastmilk, both of which are aqueous. Enzymes must be in an aqueous environment to be catalytically active^43^ and we found that EG only reacted with the pad of the strip when test samples were diluted with water. Undiluted EG has a much higher viscosity than saliva and breastmilk and does not absorb well into the pad of the strip to facilitate the enzymatic reaction unless diluted with water before application to the pad. Neat syrups spiked with EG successfully worked without the need to dilute in water (Fig. 6) since syrups already contain water. However, the water content of neat syrups is low, compared to saliva/breastmilk for which the strips are intended for, and therefore sample absorption into the pad was found to be poor. We found that a 5× dilution in water worked best to help syrup absorption into the pad. This dilution with water adds only very minimal human manipulation compared to the relatively more complicated protocols for TLC and GC. Human manipulation could easily be reduced further if the test was developed into a kit which comprises of a strip and a tube with pre-aliquoted volume (e.g. 0.95 mL) of water. In this case, the inspector would only need to add 50 mg (approx. 50 µL which is a droplet) of the raw ingredient to achieve the same 5% *v/v* as we used in our study. If the end user does not have access to pipettes or a kit, then simply five drops of the raw ingredient (approx. 250 mg) could be mixed with a teaspoon of water (approx. 5 ml) to achieve the same 5% *v/v* sample to test using the strips (see Supplementary Protocol). In either case, human manipulation is minimal and would not affect determining presence of EG.

As expected, ethanol-containing syrups interfered with both the strips and ethanol kit used in this study since ethanol is the preferred substrate for alcohol oxidase and alcohol dehydrogenase (Supplementary Fig. 3 and Fig. 6). However, ethanol is rarely used in modern paediatric syrups, and to our knowledge, all known infant fatalities were due to DEG/EG contaminated syrups which did not contain ethanol.

The strips would be useful for syrup manufacturers for additional testing of incoming raw materials and checking for EG barrels which have been mislabelled, such as the reported incidents in Indonesia^42^ and Pakistan^19^, where barrels containing 96–99%^42^ and up to 100% EG^19^, respectively, were mislabelled as PG.

The rapid alcohol test strips were able to determine levels as low as 0.5% m/m EG in PG (Fig. 5b). While this does not meet the 0.1% m/m International and United States Pharmacopeia regulatory threshold, it may help to prevent deaths and/or toxicity. This is only the case if 0.5% m/m is below the fatal/toxic concentration limits although there is no published evidence on these clinical limits. It may be possible to improve the sensitivity down to 0.1% m/m if a more sensitive fluorogenic substrate can be employed in future studies. The strips were less sensitive when testing EG spiked into syrups (Fig. 7) where detection was down to approximately 1–2% EG. Albeit above the regulatory limit, it would still help in determining major EG contaminations such as those reported in Indonesia and have the potential to prevent incidences involving major toxicity and/or deaths. These test strips were only tested with glycerol and PG as excipients used in the syrups since these alcohols are commonly used in relatively large amounts and recent cases of contamination mainly involved PG^19,42^. However, the US FDA do additionally recommend testing maltitol and sorbitol solutions for DEG and EG^32^. We show that the alcohol strips show no sign of blue colour change with the maltitol-containing syrups, Benylin Infant and Calprofen, and the sorbitol-containing syrup, Dimetapp (Table 1; Fig. 6). This suggests that these test strips can not only determine EG presence in PG- and glycerol-containing medicinal products but they could also be used to determine EG presence in both maltitol and sorbitol solutions used in the manufacturing of various liquid pharmaceutical products. We have also demonstrated that the alcohol test strips could successfully determine the presence of EG down to 1% m/m in a maltitol-containing syrup, Benylin Infant (Fig. 7). Furthermore, sorbitol-containing Dimetapp did not interfere with the alcohol dehydrogenase and aldehyde dehydrogenase-based assay (Fig. 4a) or the glycolate oxidase-based assay (Fig. 4b).

In addition to the strips being low-cost, rapid and easy to use, they do not require cold storage and can be stored, purchased and used at ambient temperatures. Although the enzymes in both the ethanol and glycolic acid assays require long-term cold storage, the enzyme reactions were prepared and incubated at room temperature without the need for an oven.

Disposable breathalysers that were repurposed to determine DEG and EG (Fig. 8), contained a proprietary oxidiser which changes from white to pink/brown colour in the presence of some alcohols. Some disposable breathalysers contain potassium dichromate which is orange in colour and changes to green in the presence of alcohols. We did not test potassium dichromate due to its extreme toxicity and deemed unsafe to be used in the field to determine DEG or EG. The advantage of the disposable breathalysers employed in this study is its safety since the chemical oxidiser is contained inside the plastic case of the breathalyser which prevents direct contact with the staff running the test. DEG was less reactive to the oxidiser in the breathalyser compared to glycerol and PG, allowing DEG to be determined successfully. On the other hand, EG did oxidise but at a lower rate also facilitating its identification. While the breathalysers are low-cost (around $1) and the reaction is rapid (10 s), since a positive result was observed with glycerol and PG and the test relies on a firm negative result for DEG (and slower positive for EG), the breathalysers can be used for testing raw materials but not with finished products. Glycerol and PG oxidise to result in a dark brown colour, whereas when the rate of colour change is lower or almost none, a suspicion of probable EG or DEG contamination, respectively, could be raised. This method may be beneficial to prevent cases of DEG and EG contamination in medical products. Substandard Coldrif and Naturcold cough syrups were contaminated with 48.6% DEG in India^23^ and 28.6% DEG in Cameroon^17^, respectively. With such high contamination levels, it is highly possible that the barrels of raw material used contained neat DEG corroborating to the typical 20–30% glycerol and/or PG content in medicinal syrups. Furthermore, these breathalysers can also help in detecting unsolicited presence of EG in PG or glycerol barrels, like found in Indonesia and Pakistan where barrels illicitly contained 96–100% EG^14,42^.

Although the crystals in the breathalyser are unknown, a possible explanation could be a chemical such as potassium permanganate and potassium iodide. Potassium permanganate is usually purple but is colourless in its oxidised/acidified form which could be why the unreacted crystals appear white. After adding the alcohols, they could be reducing potassium permanganate to result in the pink colour change. Potassium iodide may be oxidised to iodine resulting in the brown colour. The ether linkage in DEG decreases the electrophilic reactivity of the hydroxyl groups and may have greater steric hindrance resulting in less accessible hydroxyl groups for reaction with potassium permanganate. This may explain why DEG is less reactive than glycerol, PG and EG which have more reactive hydroxyl groups since they are freely accessible and can donate electrons more easily. Therefore, DEG may be a weaker reducing agent compared to glycerol, PG and EG and the reason why no colour change was seen for DEG with the breathalysers. While this is a possible explanation, we cannot be certain without knowing the identity of the chemicals in the breathalysers.

We also explored the potential use of a competitive PEG ELISA in determining DEG (Supplementary Fig. 6). The antibody in the kit recognises the polyether chain of PEG and was tested to see if it could recognise the ether in DEG. There was some weak preferential binding to DEG (lowest absorbance) compared to the other alcohols although the difference was so minor that it could not be used to differentiate DEG from the other alcohols and the breathalysers were considerably better to help determine DEG. The poor performance of the PEG ELISA was not surprising since it uses an antibody specific for PEG instead of DEG. A custom antibody or aptamer against DEG, due to its ether group and differences in branching/topology, may improve binding and DEG detection. While it is possible to develop custom antibodies and aptamers for small molecules, it may be challenging to develop them to be specific for DEG, especially when levels of other alcohols such as glycerol and PG can also be present simultaneously in these syrups at much higher concentrations. But if a custom antibody can specifically recognise DEG and avoids interaction with glycerol and PG due to minor differences in branching, this can be potentially used in a rapid test. This may incur a substantial initial development cost but can produce a very low-cost test to run.

## Conclusions

Enzymatic assays are useful in determining DEG and EG in both raw materials and finished products (summarised in Table 2). By repurposing alcohol rapid test strips, we demonstrated that we can simply, inexpensively (less than $1) and rapidly (in under 2 min) determine EG presence. These strips can be used to save the lives of the hundreds of children in cases similar to Indonesia where the problem was mainly with EG contamination. The limit of detection of the repurposed assays in determining EG ranged from 0.1 to 2% m/m. Breathalysers are also simple, inexpensive (~$1) and can rapidly (in just 10 s) identify both DEG and EG mislabelling of raw materials. The novel approaches are considerably easier and safer to carry out than sophisticated GC and TLC with only a few simple steps and could therefore be performed by staff outside of any formal scientific laboratory and with minimal training.

## Methods

### Chemicals and sample preparation

Ethylene glycol (≥ 99%, Sigma-Aldrich Cat. No. 102466), diethylene glycol (for synthesis, Sigma-Aldrich Cat. No. 8.03131), propylene glycol (Ph. Eur. grade, Sigma-Aldrich Cat. No. 16033), and glycerol (Ph. Eur. grade, Sigma-Aldrich Cat. No. 49779) were used in the study. Each of these four alcohols was prepared as 5% v/v solutions by weighing 0.5 mL of the alcohols and adding ultrapure distilled water (Milli-Q, Merck Millipore) up to 10.0 mL. EG-spiked PG samples were prepared by weighing EG in an amount to generate a percentage of 10.0, 5.0, 2.0, 1.0, 0.5, 0.1, and 0.05% m/m in PG.

### Finished medical products

Eleven medicinal syrups were purchased OTC from local pharmacies in the UK, US and Indonesia (Table 1). EG-spiked samples of representative OTC syrups were prepared by gravimetrically adding EG to the syrup matrix, generating 10.0, 5.0, 2.0, 1.0, 0.5, 0.1, 0.05 and 0.01% m/m solutions. The products include adult syrups containing ethanol (Beechams, Covonia, and Benylin Chesty) and paediatric syrups without ethanol (Dimetapp, Benylin Infant’s, and Piriteze).

### Converting the alcohols with alcohol dehydrogenase and aldehyde dehydrogenase

Alcohol dehydrogenase and aldehyde dehydrogenase in an ethanol assay kit (Megazyme, Cat. No. K-ETOH, Wicklow, Ireland) were used to convert the alcohols. In a clear-flat 96-well microplate (Greiner Bio-One, Stonehouse, UK), 10 µL of the sample was mixed with 200 µL of Milli-Q water, 20 µL of buffer, 20 µL of nicotinamide adenine dinucleotide (NAD^+^), and 5 µL of alcohol dehydrogenase. A blank using 10 µL of buffer and a sample diluent control with 10 µL Milli-Q water were also prepared. The samples in the microplate were kept at ambient room temperature (RT, recorded as 20 ± 1 °C) for 2 min and the first absorbance (A1) was measured at 340 nm on a Clariostar Plus microplate reader (BMG Labtech, Ortenberg, Germany). Without delay, 2 µL of aldehyde dehydrogenase enzyme was added to the reaction mix, incubated for 5 min at RT, and measured for the second absorbance at 340 nm (A2; absorbance peak for NADH, the reduced form of NAD^+^). Absorbances were measured every minute for 15 min. The final absorbance was achieved from the subtraction of A2 and A1. The resulting absorbances were then blank-subtracted. The acids formed in the wells of the plate were then used for the glycolic acid assay.

### Glycolic acid assay

A fluorometric glycolic acid assay kit (Abcam Cat. No. 282915, Cambridge, UK) was used according to the manufacturer’s protocol. The samples used were the samples produced after using alcohol dehydrogenase and aldehyde dehydrogenase and were diluted 10× in assay buffer. These diluted samples (50 µL) were added into wells of a 96-well black plate for fluorescence with a flat bottom (Corning, UK). For each sample, a reaction mix was prepared consisting of 44 µL of assay buffer, 2 µL of detection reagent, 2 µL of enzyme mix containing glycolate oxidase and 2 µL of fluorogenic probe. This reaction mix (50 µL) was then added to each diluted sample in the 96-well black plate and the fluorescence was recorded in 30-second intervals for 90 min at RT with the excitation/emission set to 535/587 nm. The fluorescence for the blank was subtracted from the fluorescence measurements for each sample.

### Alcohol strip tests

Glycerol, PG, DEG and EG diluted in ultrapure water to 5% v/v were tested by adding 20 µL of the diluted alcohol onto the pad of three brands of alcohol rapid test strips for saliva (Surescreen Diagnostics, Eagle Park, UK; One Step distributed by Home Health UK, Bushey, UK; AllTest distributed by UK Drug Testing, Aylsham, UK) and two brands of rapid alcohol test strips for breast milk (Frida, Miami, Florida, USA; Easy@Home, Burr Ridge, Illinois, USA). Spiked samples prepared in PG and cough syrup samples were diluted in water before being added to the pad, and the best dilution was found to be a 5× dilution in Milli-Q water. After 2 min, the intensity of the resultant blue colour was analysed visually by two individuals.

### Alcohol breathalysers

Disposable breathalysers (One Step alcohol breath test manufactured by Test&Drive, Ploty, Poland and distributed by Home Health UK, Bushey, UK) were repurposed to test glycerol, PG, DEG, and EG diluted to 1% and 0.1% v/v in Milli-Q water. The protective foil of the breathalyser was pierced by pressing firmly inwards on the protective caps on both ends according to the manufacturer’s instructions. The diluted alcohols (75 µL) were pipetted into the blowing end of the tube and flicked twice to ensure that the liquid reaches the white crystals. The breathalysers were left at RT for 2 min, and any colour changes observed visually with the crystals were recorded at 10 s and 2 min by two individuals.

### Polyethylene glycol (PEG) ELISA

A competitive PEG ELISA kit (Abcam Cat. No. ab215546, Cambridge, UK) was used according to the manufacturer’s protocol. A mixture of 50 µL sample and 50 µL of 1× PEG-HRP was prepared and 50 µL of the mixture was then added to the anti-PEG antibody-coated well of a strip in the kit. The reaction was incubated for 45 min at RT on a plate shaker set to 400 rpm. Following the incubation, the sample mix was aspirated and the well was washed three times with 1× Wash Buffer. A volume of 100 µL of TMB substrate was then added to the well and incubated for 15 min at RT in the dark with shaking, prior to the addition of 100 µL of stop solution and absorbance was read at 450 nm.

### Statistical analysis

Comparisons between groups were performed using the ordinary one-way (for samples in a group) or two-way (for samples in multiple groups) ANOVA with Dunnett’s multiple comparisons and a single pooled variance. Statistical analysis was performed using GraphPad Prism v.10.1.2 (GraphPad Software, Boston, MA, USA). A *p*-value less than 0.05 was considered statistically significant.

## Supplementary Information

Below is the link to the electronic supplementary material.

Supplementary Material 1.

Supplementary Material 2.

## Data Availability

All data generated or analysed during this study are included in this published article and its supplementary information files.

## References

[1] Pyzik, O. Z. & Abubakar, I. Fighting the fakes: Tackling substandard and falsified medicines. *Nat. Rev. Dis. Primers*. **8**, 1–2 (2022). 10.1038/s41572-022-00387-1 35982063

[2] Newton, P. N. et al. Global access to quality-assured medical products: The Oxford statement and call to action. *Lancet Global Health*. **7**, e1609–e1611 (2019). 31708137 10.1016/S2214-109X(19)30426-7

[3] Medicine Quality Research Group, University of Oxford. Oxford Statement following the MQPH 2018 Conference. https://www.tropicalmedicine.ox.ac.uk/events/medicine-quality/mqph2018/oxford-statement

[4] World Health Organization. *WHO Global Surveillance and Monitoring System for Substandard and Falsified Medical Products* (World Health Organization, 2017).

[5] Kraut, J. A. & Kurtz, I. Toxic alcohol ingestions: Clinical features, diagnosis, and management. *Clin. J. Am. Soc. Nephrol.* **3**, 208–225 (2008). 18045860 10.2215/CJN.03220807

[6] Winek, C. L., Shingleton, D. P. & Shanor, S. P. Ethylene and diethylene glycol toxicity. *Clin. Toxicol.* **13**, 297–324 (1978). 737988 10.3109/15563657808988239

[7] Schep, L. J., Slaughter, R. J., Temple, W. A. & Beasley, D. M. G. Diethylene glycol poisoning. *Clin. Toxicol.* **47**, 525–535 (2009). 10.1080/15563650903086444 19586352

[8] Jacobsen, D. et al. Ethylene glycol intoxication: Evaluation of kinetics and crystalluria. *Am. J. Med.* **84**, 145–152 (1988). 3337119 10.1016/0002-9343(88)90024-1

[9] Porter, W. H., Rutter, P. W., Bush, B. A., Pappas, A. A. & Dunnington, J. E. Ethylene glycol toxicity: The role of serum glycolic acid in Hemodialysis. *J. Toxicol. Clin. Toxicol.* **39**, 607–615 (2001). 11762669 10.1081/clt-100108493

[10] Wu, A. H. B. et al. National academy of clinical biochemistry laboratory medicine practice guidelines: Recommendations for the use of laboratory tests to support poisoned patients who present to the emergency department. *Clin. Chem.* **49**, 357–379 (2003). 12600948 10.1373/49.3.357

[11] Moreau, C. L. et al. Glycolate kinetics and Hemodialysis clearance in ethylene glycol poisoning. META study group. *J. Toxicol. Clin. Toxicol.* **36**, 659–666 (1998). 9865233 10.3109/15563659809162613

[12] Sharfstein, J. M. Elixir Sulfanilamide. in *The Public Health Crisis Survival Guide: Leadership and Management in Trying Times* (ed. Sharfstein, J. M.) 10.1093/oso/9780190697211.003.0002 (Oxford University Press, 2018).

[13] World Health Organization. Medical Product Alert N°6/2022: Substandard (contaminated) paediatric medicines. (2022). https://www.who.int/news/item/05-10-2022-medical-product-alert-n-6-2022-substandard-(contaminated)-paediatric-medicines

[14] World Health Organization. Medical Product Alert N°7/2022: Substandard (contaminated) paediatric liquid dosage medicines. (2022). https://www.who.int/news/item/02-11-2022-medical-product-alert-n-7-2022-substandard-(contaminated)-paediatric-liquid-dosage-medicines

[15] World Health Organization. Medical Product Alert N°1/2023: Substandard (contaminated) liquid dosage medicines. (2023). https://www.who.int/news/item/11-01-2023-medical-product-alert-n-1-2023-substandard-(contaminated)-liquid-dosage-medicines

[16] World Health Organization. Medical Product Alert N°4/2023: Substandard (contaminated) syrup medicines. (2023). https://www.who.int/news/item/25-04-2023-medical-product-alert-n-4-2023--substandard-(contaminated)-syrup-medicines

[17] World Health Organization. Medical Product Alert N°5/2023: Substandard (contaminated) syrup medicines. (2023). https://www.who.int/news/item/19-07-2023-medical-product-alert-n-5-2023--substandard-(contaminated)-syrup-medicines

[18] World Health Organization. Medical Product Alert N°6/2023: Substandard (contaminated) syrup medicines. (2023). https://www.who.int/news/item/07-08-2023-medical-product-alert-n-6-2023--substandard-(contaminated)-syrup-medicines

[19] World Health Organization. Medical Product Alert N°1/2024: Falsified (contaminated) USP/EP PROPYLENE GLYCOL. (2024). https://www.who.int/news/item/15-04-2024-medical-product-alert-n-1-2024--falsified-(contaminated)-usp-ep-propylene-glycol

[20] World Health Organization. Medical Product Alert N°8/2023: Substandard (contaminated) syrup and suspension medicines. (2023). https://www.who.int/news/item/07-12-2023-medical-product-alert-n-8-2023--substandard-(contaminated)-syrup-and-suspension-medicines

[21] Bangkok Post. Toxin found in 15 syrup products for children. (2024). https://www.bangkokpost.com/thailand/general/2807819/toxin-found-in-15-syrup-products-for-children

[22] Singh, R. Over 100 Indian cough syrup samples fail quality tests, linked to deaths. (2024).

[23] World Health Organization. Medical Product Alert N°5/2025: Substandard (contaminated) oral liquid medicines. https://www.who.int/news/item/13-10-2025-medical-product-alert-n-5-2025--substandard-(contaminated)-oral-liquid-medicines (2025).

[24] Wasswa, H. African countries recall batch of Johnson and Johnson cough syrup because of toxicity concerns. *BMJ* **385**, q923 (2024). 38649184 10.1136/bmj.q923

[25] World Health Organization. Diethylene Glycol (DEG) and Ethylene Glycol (EG) contamination - analytical methods developed for testing paediatric medicines. (2023). https://www.who.int/news/item/01-12-2023-diethylene-glycol-(deg)-and-ethylene-glycol-(eg)-contamination---analytical-methods-developed-for-testing-paediatric-medicines

[26] Fikri, E. & Firmansyah, Y. W. A case report of contamination and toxicity of ethylene glycol and diethylene glycol on drugs in Indonesia. *Environ. Ecol. Res.* **11**, 378–384 (2023).

[27] Umar, T. P., Jain, N. & Azis, H. Endemic rise in cases of acute kidney injury in children in Indonesia and gambia: What is the likely culprit and why? *Kidney Int.* **103**, 444–447 (2023). 36639266 10.1016/j.kint.2022.12.004

[28] Drugmakers in India warned as toxic cough syrup linked to deaths of 24 children, *Sky News*, https://news.sky.com/story/drugmakers-in-india-warned-as-toxic-cough-syrup-linked-to-deaths-of-24-children-13451542 (2025).

[29] BPOM RI. Penjelasan BPOM RI NOMOR HM.01.1.2.11.22.178 Tanggal 9 November 2022 Tentang Perkembangan Hasil Pengawasan Sirup Obat dan Penindakan Bahan Baku Propilen Glikol yang Mengandung Cemaran EG dan DEG Melebihi Ambang Batas (2022).

[30] BPOM RI. Penjelasan BPOM RI Nomor HM.01.1.2.12.22.186 Tanggal 7 Desember 2022 Tentang Pencabutan Izin Edar Sirup Obat Produksi PT Rama Emerald Multi Sukses (PT REMS, 2022).

[31] Alkahtani, S., Sammons, H. & Choonara, I. Epidemics of acute renal failure in children (diethylene glycol toxicity). *Arch. Dis. Child.* **95**, 1062–1064 (2010). 21062849 10.1136/adc.2010.183392

[32] Food and Drug Administration. Testing of Glycerin, Propylene Glycol, Maltitol Solution, Hydrogenated Starch Hydrolysate, Sorbitol Solution, and other High-Risk Drug Components for Diethylene Glycol and Ethylene Glycol Guidance for Industry. (2023).

[33] Schier, J. G., Rubin, C. S., Miller, D., Barr, D. & McGeehin, M. A. Medication-associated diethylene glycol mass poisoning: A review and discussion on the origin of contamination. *J. Public. Health Policy*. **30**, 127–143 (2009). 19597445 10.1057/jphp.2009.2

[34] Waring, W. S. Poisoning by alcohols and glycols. *Medicine* **52**, 358–363 (2024).

[35] Kraut, J. A. Diagnosis of toxic alcohols: Limitations of present methods. *Clin. Toxicol. (Phila)*. **53**, 589–595 (2015). 26114345 10.3109/15563650.2015.1056880

[36] Shin, J. M., Sachs, G. & Kraut, J. A. Simple diagnostic tests to detect toxic alcohol intoxications. *Transl Res.* **152**, 194–201 (2008). 18940722 10.1016/j.trsl.2008.07.002 PMC2615242

[37] Altamimy, M. A. et al. A selective gas Chromatography–Tandem mass spectrometry method for quantitation of ethylene and diethylene glycol in paediatric syrups. *Heliyon* **10**, e27559 (2024). 38560135 10.1016/j.heliyon.2024.e27559 PMC10980933

[38] Jähnke, R. W. O. & Dwornik, K. A. Concise quality control guide on essential drugs and other medicines: Special edition 2024 for the testing of toxic impurities in liquids for oral use. (2024).

[39] Singh, J. et al. Diethylene glycol poisoning in Gurgaon, India, 1998. *Bull. World Health Organ.* **79**, 88–95 (2001). 11242827 PMC2566350

[40] World Health Organization. Tests for diethylene glycol and ethylene glycol in liquid preparations for oral use. Chapter for inclusion in The International Pharmacopoeia. (2023).

[41] Bhakta, H. C., Choday, V. K. & Grover, W. H. Musical instruments as sensors. *ACS Omega*. **3**, 11026–11032 (2018). 30288461 10.1021/acsomega.8b01673 PMC6166230

[42] Widianto, S. Deadly Indonesian cough syrup was almost pure toxin, court papers show. (2023). https://www.reuters.com/business/healthcare-pharmaceuticals/deadly-indonesian-cough-syrup-was-almost-pure-toxin-court-papers-show-2023-10-13/

[43] Zaks, A. & Klibanov, A. M. The effect of water on enzyme action in organic media. *J. Biol. Chem.* **263**, 8017–8021 (1988). 3131337

