# Supplementary material for: Rapid screening of ethylene glycol and diethylene glycol in raw materials and medicinal syrups using low-cost field deployable assays
Source: Sci Rep. 2025 Dec 3;15:39737. doi: 10.1038/s41598-025-26670-1 (PMC12675669; doi:10.1038/s41598-025-26670-1)
Supplement: Supplementary file 1 — Supplementary Material 1. [file 41598_2025_26670_MOESM1_ESM.pdf]

Reference: Arman *et al.*, (2025), Rapid screening of ethylene glycol and diethylene glycol contamination in raw materials and medicinal syrups using simple, low-cost and field deployable assays, *Scientific Reports*.

UNIVERSITY OF OXFORD

Determining ethylene glycol (EG) presence using alcohol test strips

In raw material

The test will work for EG barrels either mislabelled as PG or glycerol or PG and glycerol barrels with > 0.5% EG contamination.

Step 1

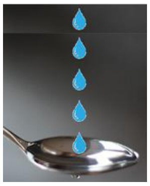

Add five drops of the raw material to a teaspoon (5 mL) of water. Mix well.

Step 2

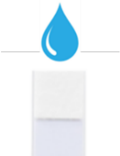

Add one drop of the diluted raw material (from Step 1) to the pad of the test strip.

Step 3

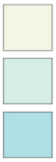

Note colour change of the pad after 2 minutes.

In finished products

The test can successfully determine the presence of EG in finished products provided the following conditions are met:

- The product does not contain ethanol as an ingredient (unless the level is so low that it does not interfere with the assay)
- The test relies on colour change due to a chemical reaction and therefore the product colour should be light enough to not mask the test results
- A known standard product (EG-free) is also tested for comparison for validity of the test results

Step 1

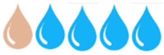

Add one drop of syrup to 4 drops of water. Mix well.

Step 2

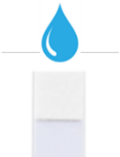

Add one drop of the diluted syrup (made up in Step 1) to the pad of the test strip.

Step 3

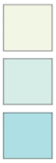

Note any colour change of the pad at 2 minutes.

Expected results:

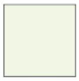

No change in colour indicates that EG is not present or <0.5%

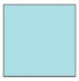

Change to blue colour indicates presence of EG (0.5% or higher).
The darker the colour, the higher the % of EG present

Reference: Arman *et al.*, (2025), Rapid screening of ethylene glycol and diethylene glycol contamination in raw materials and medicinal syrups using simple, low-cost and field deployable assays, *Scientific Reports*.

UNIVERSITY OF OXFORD

Differentiating the presence of diethylene glycol (DEG) and ethylene glycol (EG) from propylene glycol and glycerol using breathalysers

The test will work for DEG or EG barrels mislabelled as propylene glycol or glycerol.

The test can only be used for raw material and cannot be used for finished products.

Step 1

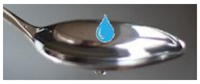

Add one drop of the raw material to a teaspoon (5 mL) of water. Mix well.

Step 2

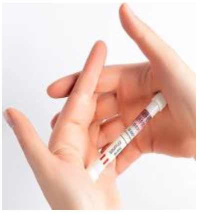

Take the breathalyser out of the packaging and press firmly on both ends of the tube to break the seal.

Step 3

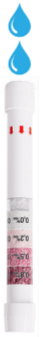

Add 2 drops of the diluted raw material (from Step 1) to the end of the breathalyser marked with the downward red arrows.

Hold the end with the red arrows and flick twice to help the liquid reaching white crystals present in the middle of the breathalyser.

Step 4

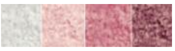

Note any colour change of the white crystals after 10 seconds to 2 minutes.

Expected results:

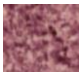

Dark brown expected for propylene glycol and glycerol from 10 seconds onwards

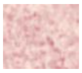

Pink seen for EG after 10 seconds (but develops into dark brown after 2 minutes)

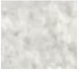

No change in colour for DEG even after 2 minutes

<u>Disclaimer</u>: These protocols are provided as supplementary materials in the best interest of the public for use by staff in the pharmaceutical industry, supply chain, or regulatory and other health agencies. Please refer to the full manuscript for the chemical principle behind the test, limitations of assay, and assumptions used. All results must be interpreted with caution. These tests should only be used for rapid screening as a guide, and the barrels or lots of raw material identified as contaminated must be verified by compendial tests for final results.
