# Supplementary material for: Rapid screening of ethylene glycol and diethylene glycol in raw materials and medicinal syrups using low-cost field deployable assays
Source: Sci Rep. 2025 Dec 3;15:39737. doi: 10.1038/s41598-025-26670-1 (PMC12675669; doi:10.1038/s41598-025-26670-1)
Supplement: Supplementary file 2 — Supplementary Material 2. [file 41598_2025_26670_MOESM2_ESM.docx]

**Supplementary Information for Arman BY, *et al.* Rapid screening of ethylene glycol and diethylene glycol in raw materials and medicinal syrups using low-cost field deployable assays (2025).**


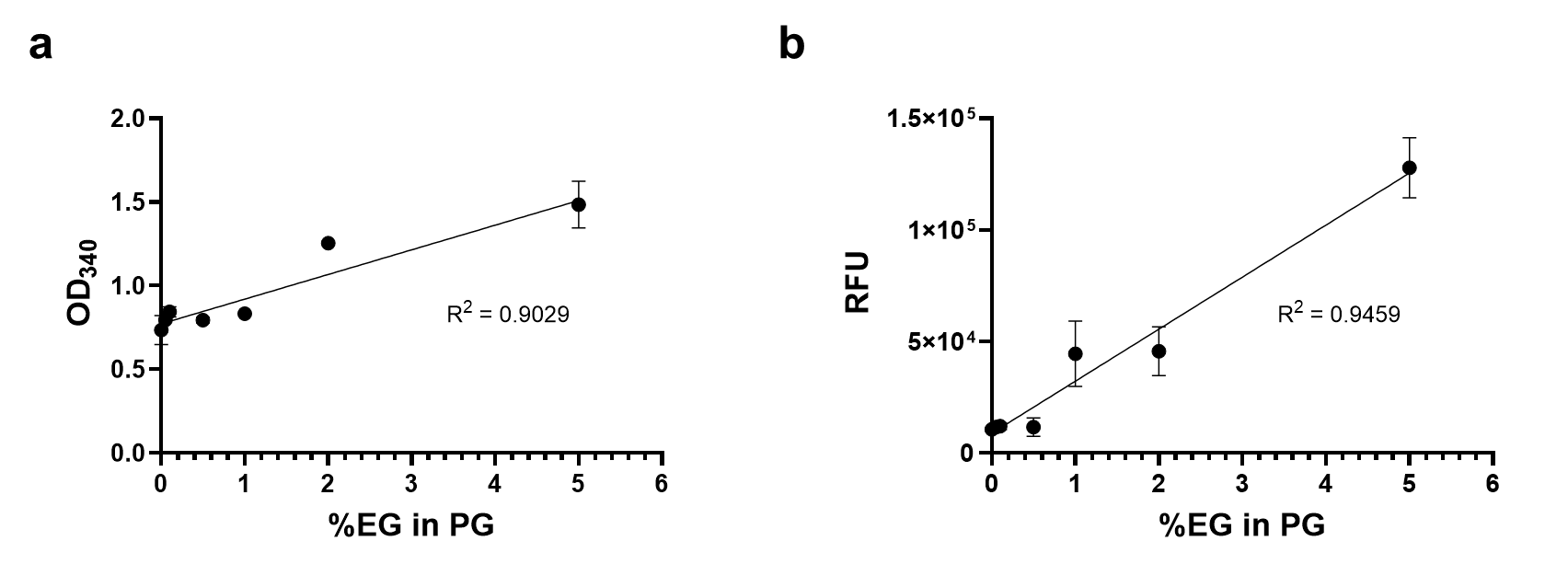


**Supplementary Fig. 1. Linear regression analysis of different percentages of EG in PG using enzymatic assays.** **a** End-point 340 nm absorbance readings of NADH after using alcohol dehydrogenase and aldehyde dehydrogenase. R-squared: 0.9029, *p*<0.0001. **b** Relative fluorescence unit (RFU) readings after additionally using glycolate oxidase in the glycolic acid assay. Error bars show the standard deviations of two replicates. R-squared: 0.9459, *p*<0.0001.


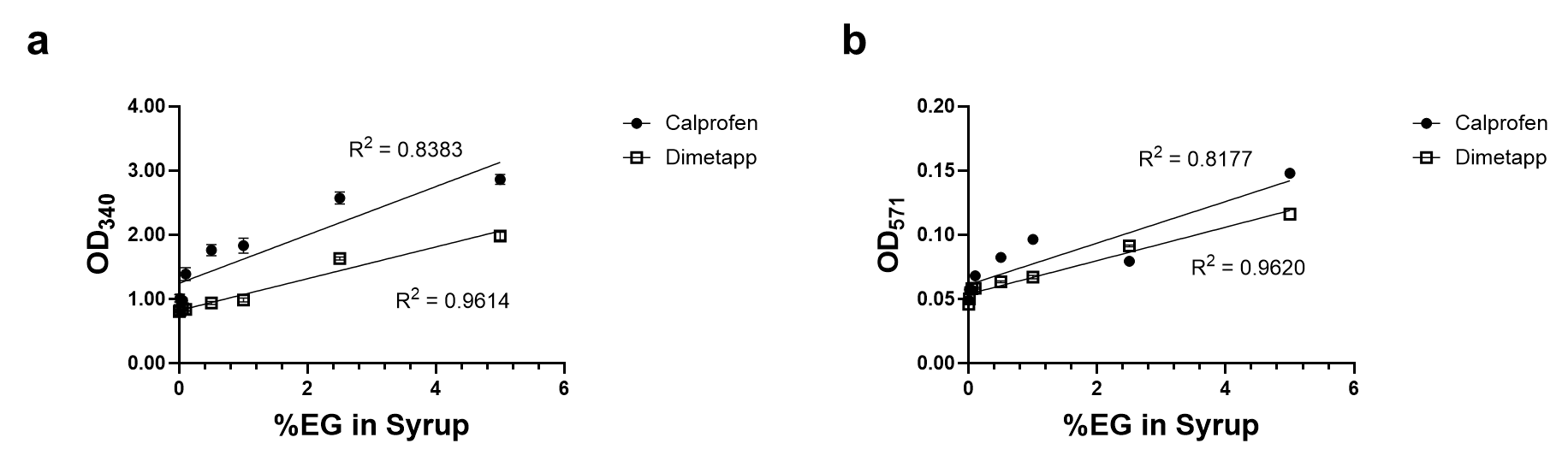


**Supplementary Fig. 2. Linear regression analysis for two paediatric syrups (Calprofen and Dimetapp) spiked with different percentages of EG.** **a** End-point 340 nm absorbance readings of NADH after using alcohol dehydrogenase and aldehyde dehydrogenase. *p*<0.0001. **b** End-point 571 nm absorbance readings after additionally using glycolate oxidase in the glycolic acid assay. Error bars show the standard deviations of two replicates. *p*<0.0001.


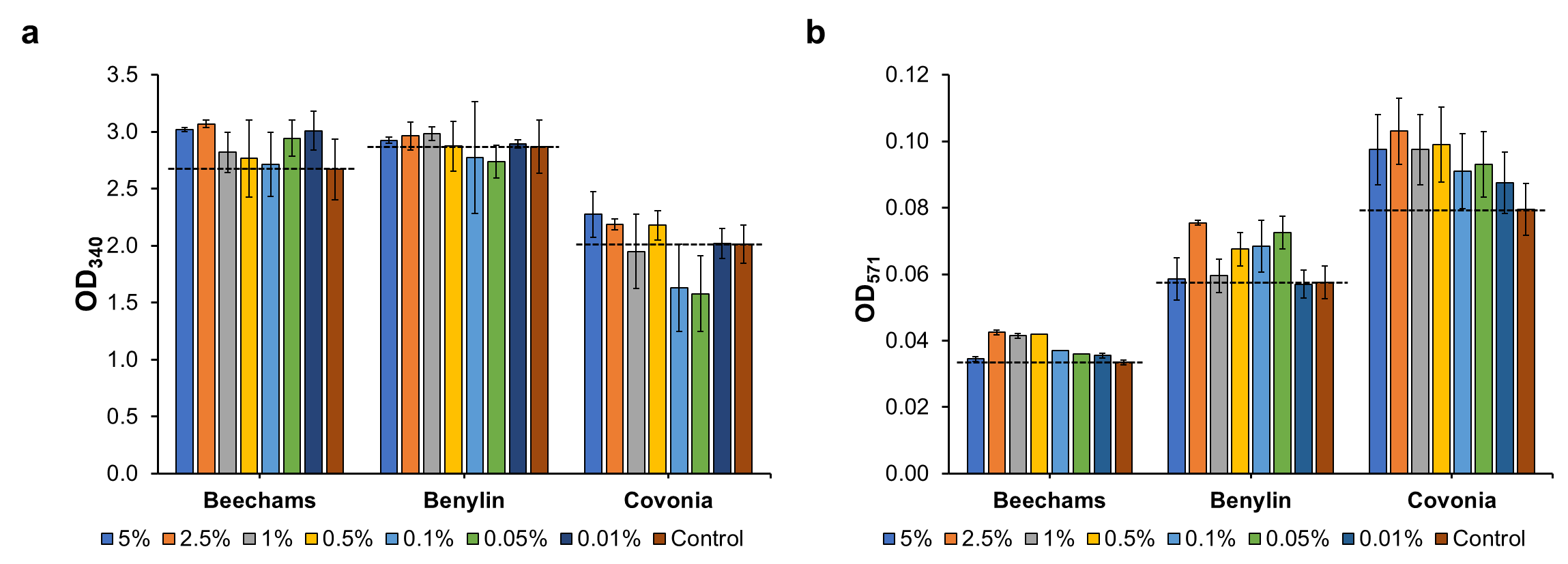


**Supplementary Fig. 3. Enzymatic assay results of three ethanol-containing medicinal syrups (Beechams, Benylin Chesty, and Covonia), spiked with different percentages of EG.** **a** End-point 340 nm absorbance readings of NADH after using alcohol dehydrogenase and aldehyde dehydrogenase. **b** End-point 571 nm absorbance readings after additionally using glycolate oxidase in the glycolic acid assay. The neat syrups without EG spiking (0% EG) were used as controls. Error bars show the standard deviations of two replicates.


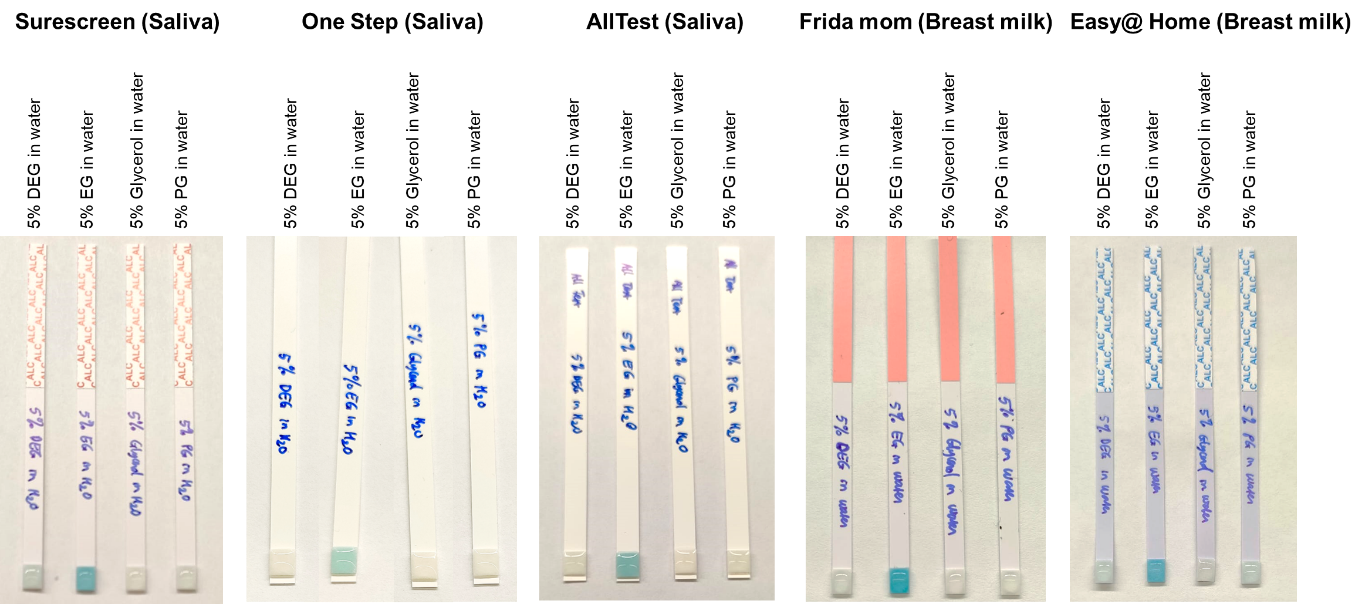


**Supplementary Fig. 4. Successful differentiation of EG from glycerol, PG and DEG using five different brands of alcohol saliva and breast milk test strips.** The best sensitivity for EG was observed using the Surescreen and Frida mom alcohol test strips although all brands could determine EG from the other alcohols with 100% accuracy. A sixth brand (Wondfo One Step Alcohol Saliva Test, Guangzhou, China) was also tested which showed similar results (data not shown).


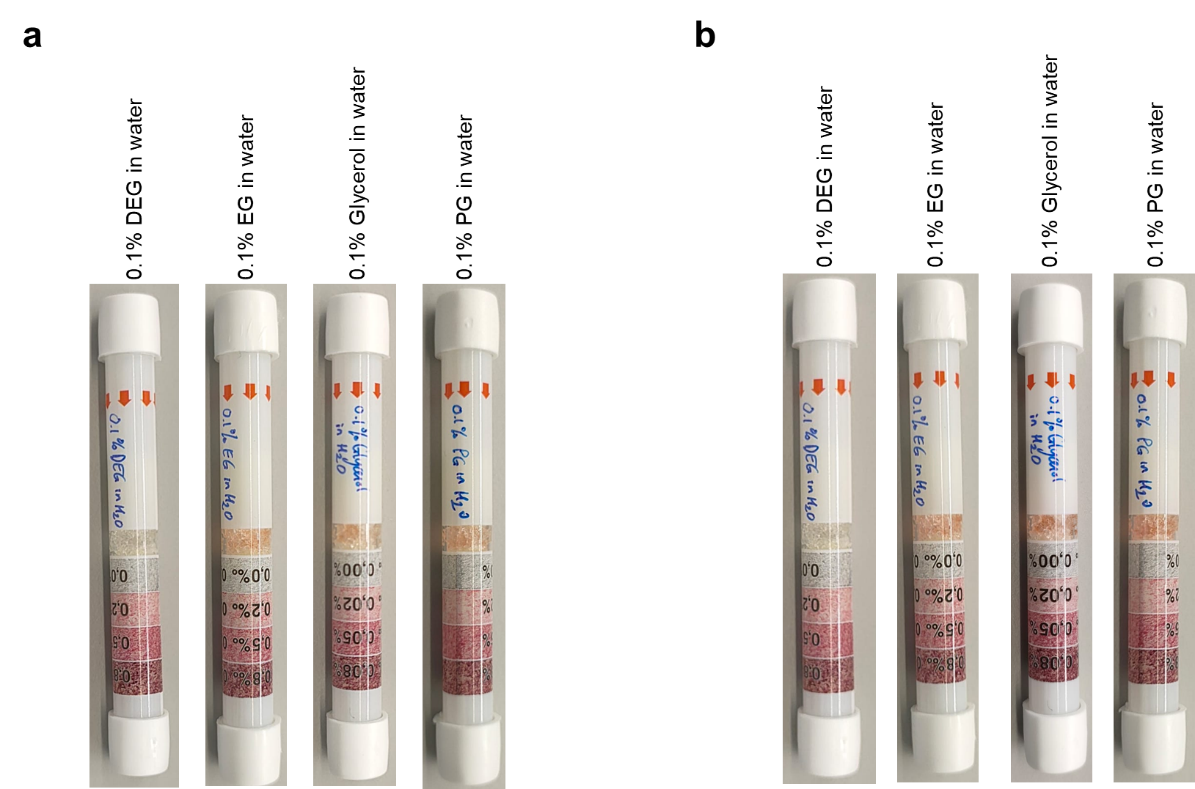


**Supplementary Fig. 5. Disposable breathalysers after testing 0.1% v/v alcohols diluted in water.** **a** Photos taken after 10 seconds and **b** after two minutes.


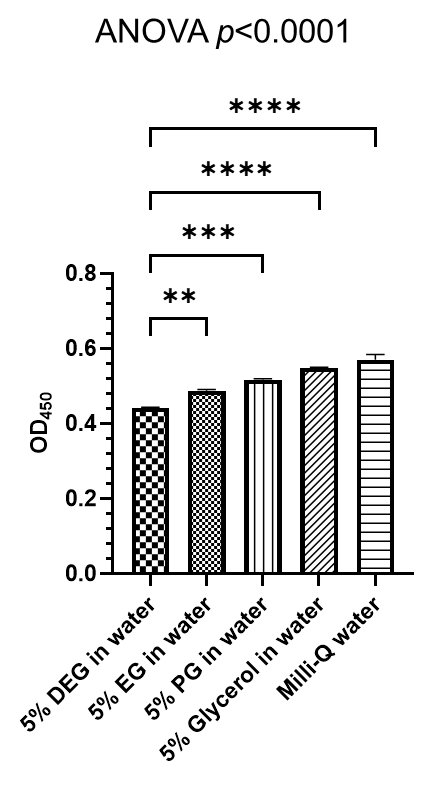


**Supplementary Fig. 6. PEG ELISA results of the alcohol solutions show very weak preferential binding to DEG.** Lower absorbances show greater binding due to this being a competitive ELISA. Error bars show the standard deviations of two replicates. Ordinary one-way ANOVA with Dunnett’s multiple comparisons were applied. **p<0.01; ***p<0.005; ****p<0.0001
